# Long-term Patient-reported Quality of Life and Pain After a Multidisciplinary Clinical Pathway for Elderly Patients With Hip Fracture: A Retrospective Comparative Cohort Study

**DOI:** 10.1177/2151459319841743

**Published:** 2019-06-06

**Authors:** Pishtiwan H. S. Kalmet, Stijn G. C. J. de Joode, Audrey A. A. Fiddelers, Rene H. M. ten Broeke, Martijn Poeze, Taco Blokhuis

**Affiliations:** 1 Department of Trauma Surgery, Maastricht University Medical Center, Maastricht, The Netherlands; 2 Nutrim School for Nutrition, Toxicology and Metabolism, Maastricht University, Maastricht, The Netherlands

**Keywords:** hip fractures, long-term patient-reported outcome, elderly, multidisciplinary clinical pathway

## Abstract

**Introduction:**

There is an increase in incidence of hip fractures in the ageing population. The implementation of multidisciplinary clinical pathways (MCP) has proven to be effective in improving the care for these frail patients, and MCP tends to be more effective than usual care (UC). The aim of this study was to analyze potential differences in patient-reported outcome among elderly patients with hip fractures who followed MCP versus those who followed UC.

**Materials and Methods:**

This retrospective cohort study included patients aged 65 years or older with a low-energy hip fracture, who underwent surgery in the Maastricht University Medical Center, Maastricht, the Netherlands. Two cohorts were analyzed; the first one had patients who underwent UC in 2012 and the second one contained patients who followed MCP in 2015. Collected data regarded demographics, patient-reported outcomes (Short Form 12 [SF-12] and the Numeric Rating Scale [NRS] to measure pain), and patient outcome.

**Results:**

This cohort study included 398 patients, 182 of them were included in the MCP group and 216 were in the UC group. No differences in gender, age, or American Society of Anesthesiologists classification were found between the groups. No significant differences were found in SF-12 and the NRS data between the MCP group and UC group. In the MCP group, significantly lower rates of postoperative complications were found than in the UC group, but mortality within 30 days and one year after the hip fracture was similar in both groups.

**Discussion:**

Although the effects of hip fractures in the elderly on patient-reported outcome, pain and quality of life have been addressed in several recent studies, the effects of MCP on long-term outcome was unclear.

**Conclusion:**

A multidisciplinary clinical pathway approach for elderly patients with a hip fracture is associated with a reduced time to surgery and reduced postoperative complications, while no differences were found in quality of life, pain, or mortality.

## Introduction

The ageing population is growing rapidly, and as a pertaining issue, the incidence of hip fractures among elderly patients is increasing as well.^1,2^ The high rate of comorbidities in this frail group is associated with high mortality rates.^3-6^ Moreover, both the functional outcome and life expectancy decrease in elderly patients with hip fractures. Several studies have shown a decrease in quality of life, mobility, and ability to perform activities of daily living among this population.^7-11^ Implementation of a multidisciplinary clinical pathway (MCP),^12^ which has been developed to optimize medical care in various patient groups,^13-19^ is one of the few effective measures to improve the outcome. In fact, the usage of MCP for elderly patients with hip fractures tends to be more effective than usual care (UC),^20,21^ resulting in lower rates of postoperative complications and a decrease in the 30-day mortality rate.^22-24^

Although the effect of MCP on postoperative complications and mortality was established before, the comparison between UC and MCP on patient-reported outcome (patient-reported quality of life and pain) has not been studied. The aim of this study was therefore to compare patient-reported outcome in elderly patients with a surgically treated hip fracture following UC versus elderly patients with a surgically treated hip fracture following MCP.

## Materials and Methods

This retrospective cohort study included patients aged 65 years or older with a surgically treated low-energy proximal femur fracture (femoral neck) and or pertrochanteric fractures (AO/OTA type 31 A [trochanteric fracture] and 31 B [femoral neck fracture]) who underwent surgery in the Maastricht University Medical Center, Maastricht, the Netherlands. Patients with a high-energy hip fracture (defined as motor vehicle and motorcycle accidents, a collision at a moped or bicycle >35 km/h, pedestrian struck by a motor vehicle >10 km/h, and fall from 2 times the body height), patients with an AO/OTA type 31 C (femoral head) proximal femoral fracture, patients with >2 fractures, and patients not living in the hospital area were excluded. Data from both cohorts were separately collected and analyzed. The first cohort regarded the data of all patients treated during the year 2012, before implementation of MCPs. This cohort is therefore referred to as the cohort submitted to UC. The second cohort regarded the data of all patients treated during the year 2015, which is 2 years after the implementation of MCP. Therefore, this cohort is referred to as the MCP cohort. Surgical treatment was performed according to the Dutch Guidelines.^25^

Usual care protocol includes standard traditional treatment by an orthopedic trauma surgeon at the trauma unit with a follow-up at the out-patient clinic. Physiotherapy is prescribed when the patient is discharged home. Multidisciplinary clinical pathways address the management of care that patients need from arrival in the emergency department until they are discharged to the rehabilitation unit or a nursing home. The multidisciplinary team consists of an orthopedic trauma surgeon, a geriatrician, an anesthesiologist, and a physiotherapist. These disciplines are all actively involved in the decision making process regarding the care that patients need from the first presentation at the emergency department until they are discharged from the hospital. Additional medical specialties remain available for consultation depending on the comorbidities of the patient. The aim of the team is to perform surgical treatment within 24 hours upon admission and to achieve discharge within 4 days. To achieve this goal, agreements have been set in place with rehabilitation facilities to transfer the surgically treated patients to a patient-centered destination as soon as possible. This may either be a rehabilitation center or nursing home with rehabilitation facilities. The postoperative protocol for both groups, MCP and UC, was early mobilization and early full weight bearing.

Data were retrospectively collected from the medical records by 2 independent researchers. Demographics included age at time of fracture, gender, ASA (American Society of Anesthesiologists; assessing the fitness of patients before surgery, type 1–6),^26^ Charlson-comorbidity score (classifying prognostic comorbidity, a higher score represents additional comorbidities),^27^ time to operating theatre (in hours), type of fracture (femoral neck fracture or pertrochanteric fracture), type of surgical procedure (prosthesis, intramedullary nail or dynamic hip screw), and length of stay (in days). Furthermore, patient-reported questionnaires were sent by mail to all surviving individuals after a minimum of 2 years to follow-up on their quality of life and pain. To ensure a sufficient response rate, all eligible participants were contacted by telephone prior to sending the questionnaires by regular mail. During this telephone call, informed consent was obtained. If the questionnaires were not returned within 30 days after sending, a new telephone call followed to improve participation.

The primary outcome measure was the patient-reported outcome questionnaire, which included 2 items, the quality of life and pain. The quality of life was measured with the Short Form 12 (SF-12).^28^ The SF-12 consists of 12 items that assess 8 dimensions of health: physical functioning, role-physical, bodily pain, general health, vitality, social functioning, role-emotional, and mental health. The SF-12 measures various aspects of physical and mental health from which physical composite score (PCS) and mental composite score (MCS) can be calculated, ranging from a minimum of 0 to a maximum of 100. The intensity of pain was measured with the Numeric Rating Scale (NRS; 0 is no pain and 10 is worst pain).^29^

The secondary outcome parameters were complications, delirium, the 30-day mortality, and one-year mortality rate. Postoperative complications (eg, complications related to the fracture and general complications not related to the fracture) were defined as any adverse event that required intervention; these were recorded as either present or non-present. Data on the occurrence of postoperative delirium were collected separately.

The medical ethics committee of Maastricht University Medical Center, Maastricht, the Netherlands approved this study and informed consent for sending the questionnaire was given by all patients.

### Statistical Analysis

Statistical analysis was performed with IBM SPSS Statistics (Version 23.0, Armonk, NY). Descriptive statistics were used to describe the demographic data and baseline characteristics for the entire study population. Independent samples *t*-tests were used for normally distributed continuous data and χ^2^ tests for categorical variables. Results are presented as either mean (standard deviation) or as frequencies and percentages. In case of non-parametric data, the median with the interquartile range are described. The level of statistical significance was set at an α of .05.

## Results

### Baseline Characteristics

This cohort study included 398 patients, 216 in the UC group and 182 in the MCP group. Patients in the UC group had more comorbidities (mean Charlson score; 7.0 [2.6] vs 6.1 [1.9], respectively, *P* < .01) and were more likely to have a femoral neck fracture than those in the MCP group. No differences in gender, age, or ASA classification were found between the groups (*P* < .05). Characteristics of patients in the MCP and UC groups are summarized in Table 1.

**Table 1.** Baseline Characteristics of the MCP and UC Groups.

	MCP (N = 182)	UC (N = 216)	Total (N = 398)	*P*
Female	129 (70.9%)	153 (70.8%)	282 (70.9%)	.99
Mean age (SD), years	83.4 (7.4)	82.2 (7.5)	82.7 (7.5)	.14
ASA				
I, II	81 (44.5%)	87 (40.3%)	168 (42.2%)	.42
III, IV	101 (55.5%)	129 (59.7%)	230 (57.8%)	
Mean Charlson score (SD)	6.1 (1.9)	7.0 (2.6)	6.6 (2.4)	<.01
Fracture type				
Femoral neck	89 (48.9%)	156 (72.2%)	245 (61.6%)	<.01
Pertrochanteric	93 (51.1%)	60 (27.8%)	153 (38.4%)	

Abbreviation: ASA, American Society of Anesthesiologists; MCP, multidisciplinary clinical pathway; SD, standard deviation; UC, usual care.

### In-Hospital Outcome

The mean time to surgery was significantly shorter in the MCP group than in the UC group: 18.2 (9.3) hours versus 25.3 (13.9) hours (*P* < .01). The number of patients who had to wait more than 24 hours was also significantly lower in the MCP group than in the UC group: 17.6% versus 44.9% (*P* < .01). There was a significant difference in the type of surgical procedures performed in the MCP versus UC groups, with more prostheses and intramedullary nails in the UC group for femoral neck and pertrochanteric fractures, respectively (Table 2).

**Table 2.** Surgical Procedures in the MCP and UC Groups.

	MCP (N = 182)	UC (N = 216)	Total (N = 398)	*P*
Femoral neck fracture				
Prosthesis	83 (45.6%)	95 (44.0%)	178 (44.7%)	<.01
Pertrochanteric fracture				
Intramedullary nail	81 (44.5%)	103 (47.7%)	184 (46.2%)	<.01
Dynamic hip screw	18 (9.9%)	18 (8.3%)	36 (9.1%)	<.01
Mean time to surgery (in hours), (SD)	18.2 (9.3)	25.3 (13.9)	22.1 (12.5)	<.01

Abbreviations: MCP, multidisciplinary clinical pathway; UC, usual care.

The mean length of hospitalization was significantly shorter in the UC group compared to the length of hospitalization in the MCP group: 12.3 (7.3) days versus 15.1 (15.7) days (*P* = .02).

### Patient-reported Outcome

After at least 2 years follow-up, only 49.9% of the patients were still alive. Fourteen of these remaining 159 patients were unable to fulfill the questionnaire due to cognitive impairment. The final overall response rate of the patient-reported questionnaire (SF-12 and the NRS) was 65.6% (95 of 145 participants). The response rate was similar in the MCP group and the UC group (69% vs 60.7%, respectively, *P* = .30). The patient-reported outcome as measured with the SF-12 showed similar scores for both the MCP and the UC cohort. Quality of life, also measured with the SF-12, and pain, measured with the NRS, were also similar for both cohorts (Table 3).

**Table 3.** Patient-reported Outcome Measurements in the MCP and UC Groups.

	MCP (N = 58)	UC (N = 37)	Total (N = 95)	*P*
Mean SF-12 (quality of life), (SD)	47.9 (24.4)	45.4 (27.6)	46.9 (25.7)	.65
PCS	36.3 (28.6)	35.8 (28.9)	36.1 (28.7)	.93
MCS	59.5 (25.0)	54.9 (31.0)	57.6 (27.5)	.45
Mean NRS (pain), (SD)				
30-day after hip fracture	4.2 (2.8)	3.9 (2.7)	4.1 (2.8)	.56
1-year after hip fracture	2.7 (2.8)	1.9 (2.7)	2.4 (2.8)	.17
At follow-up questionnaire	2.8 (4.0)	1.8 (2.7)	2.5 (3.6)	.19

Abbreviations: MCP, multidisciplinary clinical pathway; MCS, mental composite score; PCS, physical composite score; SD, standard deviation; UC, usual care.

### Complications and Mortality Outcome Measures

Postoperative complications, defined as complications requiring intervention, were common in the overall study population (65.6%). The incidence of postoperative complications was significantly lower in the MCP group (45.1%) compared to the UC group (82.9%, *P* < .01; Table 4). Postoperative delirium occurred less frequently in the MCP group than in the UC group (19.2% vs 45.4%; *P* < .01). Mortality rate within 30 days and one year after admission was 8.0% and 35.4%, respectively. The difference between the MCP group and the UC group was not significant.

**Table 4.** Patient Outcome Measurements in the MCP and UC Groups.

	MCP (N = 182)	UC (N = 216)	Total (N = 398)	*P*
Total postoperative complications	82 (45.1%)	179 (82.9%)	261 (65.6%)	<.01
Postoperative delirium	35 (19.2%)	98 (45.4%)	133 (33.4%)	<.01
Mortality				
30-day	15 (8.2%)	17 (7.9%)	32 (8.0%)	.90
1-year	61 (33.5%)	80 (37.0%)	141 (35.4%)	.50

Abbreviations: MCP, multidisciplinary clinical pathway; UC, usual care.

## Discussion

Although the effects of hip fractures in the elderly on patient-reported outcome, pain, and quality of life have been addressed in several recent studies,^23,30-33^ the effects of MCP on long-term outcome was unclear. This retrospective cohort study found that the use of MCP for elderly patients with a hip fracture was associated with a reduced time to surgery and reduced postoperative complications. Nonetheless patient-reported long-term quality of life and pain are similar for patients who are treated according to MCP or UC. Surprisingly, neither the 30-day nor the 1-year mortality rate was affected by the implementation of MCP.

The reduced time to surgery that was seen in our study was also found by other authors.^19,34,35^ This reduced time to surgery was associated with a significant reduction of the complication rate in our study. These findings are in line with several studies that described a significant increase in complications after 24 hours^36,37^ and even a significant increase in mortality after 48 hours.^38^ Although the reduced time to surgery in our study showed an effect on the complication rate, an effect on neither the 30-day nor the one-year mortality was seen.

A postoperative complication rate up to 59% has been reported in elderly people with hip fractures.^17,19,34,39^ We found comparable rates (45%) of postoperative complications in the MCP group, and the complication rate was lower than that in the UC group. This finding is in line with 2 other studies that reported similar differences.^20,40^ Delirium was scored as a separate complication in our study, since it is the most common complication in elderly patients with hip fractures. In our study, the overall incidence of delirium was 33.4%, but the incidence was significantly lower in the MCP group. This could be a direct result of the MCP approach, in which a geriatrician is consulted to impose preventive measures in each patient.

The MCP approach aims to shorten the length of stay, but our study shows a significantly longer length of stay in the MCP group compared to the UC group. This is contradictory to all other studies regarding MCP, that have shown a significant reduction in length of stay.^17-19,34,35,39,41,42^ A possible explanation for this difference is the discharge destination of the patients. Instead of going home, most patients are transferred to geriatric rehabilitation. This makes the discharge date dependent on availability of such a center and this delays discharge in many cases. The MCP for elderly patients with hip fractures might be beneficial and cost-effective regarding the hospital care, as the significant reduction in complications could make the MCP cost-effective. However, from the patient perspective, the MCP has little benefit, as no significant differences in patient-reported outcome and observed mortality rate.

In the interpretation of our data, some limitations have to be taken into account. The retrospective nature of the study limits the data quality and the use of a questionnaire induces a risk of selection bias. However, the retrieved data were found to be well documented. The response rate for the patient-reported outcome, which included the SF-12, was substantial and comparable in both groups, making the comparison between both groups in our opinion justified. It is evident that a prospective cohort study in elderly patients with hip fractures is needed to address the (cost-)effectiveness, the functional outcome, and long-term patient-reported outcome of the MCP strategy. The main practical concern of this study regarded the long period to follow-up with this frail patient group.

## Conclusion

Although this retrospective comparative cohort study shows that the MCP approach for elderly patients with a hip fracture is associated with a reduced time to surgery and reduced postoperative complications, no differences were found in long-term patient-reported quality of life or pain. Moreover, there was no significant difference in 30-day and one-year mortality.
